# HUC-MSC secretome and Nanoemulsion Propolis synergistically modulate inflammatory responses in hyperglycemia-induced calvarial osteolysis

**DOI:** 10.1016/j.jobcr.2026.101430

**Published:** 2026-03-02

**Authors:** Putri Cahaya Situmorang, Helen Helen, Syafruddin Ilyas, Salomo Hutahaean, Doni Aldo Samuel Siahaan, Ananto Ali Alhasyimi, Muh Ade Artasasta, Wibi Riawan, Khairul Anuar Shariff, Alexander Patera Nugraha

**Affiliations:** a Department of Biology, Faculty of Mathematic and Natural Science, Universitas Sumatera Utara, Medan, Indonesia; b Department of Pharmacology, Faculty of Pharmacy, Universitas Sumatera Utara, Medan, Indonesia; c Department of Orthodontic, Faculty of Dentistry, Universitas Gadjah Mada, Yogyakarta, Indonesia; d Department of Biotechnology, Faculty of Mathematic and Natural Science, Universitas Negeri Malang, Malang, Indonesia; e Department of Biochemistry and Molecular, Biochemistry Biomolecular Laboratory, Faculty of Medicine, Universitas Brawijaya, Surabaya, Indonesia; f School of Materials and Mineral Resources Engineering, Engineering Campus, Universiti Sains Malaysia, 14300, Nibong Tebal, Penang, Malaysia; g Department of Orthodontic, Faculty of Dental Medicine, Universitas Airlangga, Surabaya, Indonesia

**Keywords:** HUC-MSC, Hyperglycemia, Osteolysis, Inflammation, Cytokines, HSP-70

## Abstract

**Background:**

Hyperglycemia-induced osteolysis is a significant consequence of diabetes mellitus, marked by heightened inflammatory responses, augmented osteoclastogenesis, and compromised osteoblast function. Innovative therapy approaches aimed at inflammatory and cytoprotective mechanisms are essential to avert diabetic bone loss.

**Objective:**

This study investigated the synergistic effects of human umbilical cord mesenchymal stem cell (HUC-MSC) secretome and Nanoemulsion Propolis (NEP) on inflammatory responses and protective protein expression in a rat model of hyperglycemia-associated calvarial osteolysis.

**Methods:**

Male Wistar rats were categorized into seven groups: control, LPS-induced inflammation, hyperglycemia, a combination of LPS and hyperglycemia, and treatment groups administered HUC-MSC secretome, NEP, or both. Cytokine concentrations (IL-1β, TNF-α, IL-10) were measured using ELISA, whilst NF-κB, IL-6, HSP-70, and HSP-10 expression in calvarial tissue was assessed by immunohistochemistry.

**Results:**

LPS, in conjunction with hyperglycemia, significantly increased pro-inflammatory cytokines and NF-κB activation, reduced IL-10 levels, and inhibited HSP-70 and HSP-10, hence worsening bone resorption. Treatment with HUC-MSC secretome or NEP alone moderately diminished inflammation, however the combined therapy markedly downregulated NF-κB, IL-1β, TNF-α, and IL-6, while reinstating IL-10 and stress proteins. These modifications diminished osteoclast activation and maintained osteoblast viability, with the most significant protective impact noted in the combination group.

**Conclusion:**

The HUC-MSC secretome and NEP collaboratively influence inflammatory pathways and reinstate protective proteins in calvarial osteolysis triggered by hyperglycemia. This dual technique presents a promising acellular and natural product-based method for addressing diabetes bone loss and associated craniofacial problems.

## 1 Introduction

Osteolysis is insufficiently characterized but often occurs as a result of various chronic illnesses, including bone infections, trauma, malignancies, and notably, uncontrolled diabetes mellitus.^1^ The high incidence of diabetes in Indonesia, ranked fifth worldwide by the International Diabetes Federation in 2021, indicates a probable increase in osteolysis cases due to chronic hyperglycemia that triggers inflammation and harms bone tissue.^2^ Furthermore, limitations in the early detection and accessibility of molecular-based regenerative therapies in various regions present osteolysis as a clinical challenge that requires a novel approach. Osteolysis, a pathological process of bone resorption, is often begun by an excessive inflammatory response. When this condition manifests in individuals with diabetes mellitus or other kinds of persistent hyperglycemia, it becomes more complex.^3^^,^^4^ Hyperglycemia is recognized to exacerbate bone deterioration by elevating pro-inflammatory cytokine expression, inducing oxidative stress, and promoting osteoclastogenesis.^3^

Hyperglycemia can provoke calvarial osteolysis through multiple molecular and cellular pathways. The principal mechanism entails the activation of inflammatory pathways, specifically via the increased expression of proinflammatory cytokines such as TNF-α, IL-1β, and IL-6.^5^ Bone resorption cells called osteoclasts are activated and differentiated by cytokines, while bone building cells called osteoblasts are inhibited in their activity.^6^ In calvarial tissue characterized by reduced thickness and lower mechanical stress stimulation, this imbalance more readily leads to a reduction in bone mass and density.^7^ Hyperglycemia markedly stimulates the NF-κB pathway, induced by oxidative stress and the accumulation of advanced glycation end products (AGEs). Upon activation of NF-κB, proinflammatory cytokines such as IL-6, IL-1β, and TNF-α are expressed at elevated levels. These cytokines subsequently promote the development and activity of osteoclasts, hence exacerbating the process of osteolysis.^8^ HSP-70 functions as a protective agent by suppressing the activation of the NF-κB pathway and stabilizing intracellular structural proteins. Furthermore, HSP-70 possesses the ability to reduce osteoblast cell death and inhibit osteoclast development through anti-inflammatory mechanisms.^9^ Hyperglycemia is associated with decreased levels of IL-10, leading to an increase in pro-inflammatory cytokines and a heightened risk of bone tissue damage. IL-10 operates by inhibiting the synthesis of IL-6, IL-1β and TNF-α, while also suppressing the activation of immune cells, including macrophages and lymphocytes.^10^ Elevated IL-10 expression may suppress osteoclastogenesis and enhance osteoblast function, potentially mitigating or delaying the advancement of osteolysis in chronic inflammatory conditions. HSP10 serves a protective role by mitigating oxidative stress and cellular inflammation.^8^ Recent studies indicate that HSP10, while less extensively researched than HSP-70, can inhibit pro-inflammatory cytokine production and enhance anti-apoptotic mechanisms, particularly in bone cells under metabolic stress.^6^

The secretome generated by human umbilical cord mesenchymal stem cells (HUCMSC) exhibits considerable therapeutic potential in obstructing the advancement of osteolysis via immunomodulation and mechanisms of bone tissue regeneration.^11^ Important for osteoclast differentiation, bioactive substances in the secretome such as IL-10, TGF-β, growth factors, and extracellular vesicles hinder the activation of inflammatory pathways like NF-κB.^10^ The HUCMSC secretome additionally promotes the expression of anti-inflammatory factors and enhances osteoblast activity, thus rebalancing the bone remodeling process disrupted by chronic inflammation.^12^ When given to patients with hyperglycemic circumstances, which worsen bone resorption, secretome produced from HUCMSC reduces oxidative stress and proinflammatory cytokines.^13^ This positions it as a promising non-cellular therapeutic candidate for addressing osteolysis resulting from metabolic stress and inflammation.^13^

Bees produce Nanoemulsion Propolis from plant resins, which contains bioactive compounds such as aromatic esters, flavonoids, and phenolic acids. These compounds are recognized for their anti-inflammatory properties beneficial to bone health 14, 15, 16. NEP inhibits osteoclastogenesis, a key process in bone resorption, by decreasing the expression of pro-inflammatory cytokines, including TNF-α and IL-1β, and by obstructing the activation of the NF-κB signaling pathway.^17^ Compounds in NEP can enhance osteoblast activity, the cells responsible for bone formation, by stimulating molecular pathways including Bone Morphogenetic Protein (BMP) and Wnt/β-catenin.^18^ In pathological conditions such as osteolysis or osteoporosis, particularly those caused by hyperglycemia or chronic inflammation, the utilization of NEP—especially in nanoemulsion form to improve bioavailability—exhibits potential as a bone-protective agent, serving to mitigate inflammation and stimulate the formation of new bone tissue.^19^ NEP exhibits physicochemical properties that are not conducive to oral or systemic therapeutic use, including low water solubility, restricted bioavailability, and compositional variability influenced by the plant source and environmental factors.^20^ Nanoemulsion technology, characterized as an oil-in-water dispersion system with particle sizes under 200 nm, has been employed to enhance the dispersion, biological penetration, and pharmacological efficacy of active compounds in NEP, thereby addressing these challenges.^21^ NEP enhance absorption rates, improve systemic distribution, and provide protection against enzymatic degradation in the gastrointestinal tract.^22^ Nanoemulsion formulations facilitate targeted delivery to inflamed or bone tissues, thereby improving the anti-inflammatory and osteoprotective effects of NEP in various pathological conditions, such as hyperglycemic osteolysis.^23^ The justification for integrating HUCMSC secretome with NEP is based on their synergistic processes. The secretome comprises a diverse range of cytokines, growth factors, and extracellular vesicles that facilitate immunomodulation and tissue healing, while NEP provides polyphenolic chemicals such CAPE and quercetin, which possess potent antioxidant and NF-κB inhibitory characteristics. Collectively, these medicines are anticipated to synergistically inhibit excessive osteoclastogenesis while bolstering osteoblast resilience under hyperglycemic conditions.

Our previous report demonstrated that the HUCMSC secretome and NEP attenuate osteoclastogenesis mainly through modulation of osteoclast-related markers (NFATc1, RANK/RANKL/OPG, sclerostin, TRAP, and cathepsin K).^24^ In the present work, we extend the analysis by evaluating inflammatory mediators (IL-1β, IL-6, TNF-α, IL-10, NF-κB) and cellular stress proteins (HSP-70 and HSP-10) under hyperglycemic osteolysis conditions.^24^ This provides broader mechanistic insight into the therapeutic synergy of this combination. By demonstrating both cytokine regulation and stress-protein restoration, our study highlights a novel mechanism of action that expands the therapeutic potential of HUCMSC secretome and NEP in diabetic bone loss models.

## 2 Material and methods

### 2.1 Chemical and reagents

The antibodies utilized for Immunohistochemistry included QED Bioscience Hsp70 Monoclonal Antibody (Catalog # 11118-100UG), Bioss HSP10 Polyclonal Antibody (Catalog # BS-7026R), Invitrogen IL-6 Monoclonal Antibody (MP5-20F3), eBioscience™ (Catalog # 14-7061-81), and Invitrogen NF-kB p65 Polyclonal Antibody (Catalog # PA5-27617). The antibodies utilized in the ELISA were the Invitrogen Rat TNF alpha ELISA Kit (Catalog # KRC3011), the Invitrogen Rat IL-1 beta ELISA Kit (Catalog # BMS6002-2), and the Invitrogen Rat IL-10 ELISA Kit (Catalog # BMS629). All products are sourced from ThermoFisher Scientific, located in Massachusetts, United States. All antibodies were utilized in accordance with the manufacturers' protocols.

### 2.2 Human umbilical cord mesenchymal stem cells secretome (HUC-MSC) metabolite preparation

The umbilical cord was obtained from a healthy donor who underwent a successful Caesarean section at the Integrated Surgical Centre RSUD Dr. Soetomo in Surabaya. The informed consent has been granted and signed by the donor. A healthy donor was selected because, according to our unpublished research, the donor's health would have an impact on the stem cells' and umbilical cord's quality.^25^

Human umbilical cord mesenchymal stem cells (HUC-MSCs) were cultured in Dulbecco's Modified Eagle Medium (DMEM/F12) supplemented with 10% fetal bovine serum (FBS) and 1% penicillin–streptomycin under standard incubation conditions (37 °C, 5% CO_2_, and 95% relative humidity). Cells at passage 4 were used for secretome collection. When the cells reached 80–90% confluency, the culture medium was removed, and the cells were gently washed twice with phosphate-buffered saline (PBS) to eliminate residual serum proteins. For secretome production, 1 × 10^6^ cells were seeded into T-75 culture flasks containing 10 mL of serum-free DMEM/F12 and incubated for 48 h at 37 °C in a humidified atmosphere with 5% CO_2_. After incubation, the conditioned medium (secretome) was carefully collected and centrifuged at 1500 rpm for 3 min at 4 °C to remove cellular debris. The supernatant was subsequently filtered through a 0.45 μm syringe-driven filter unit (Merck Millipore, Burlington, USA). The filtered secretome was further purified by dialysis using a cellulose ester dialysis membrane with a molecular weight cut-off (MWCO) of 3.5 kDa (Spectra/Por®, Spectrum Laboratories, USA). Dialysis was performed against sterile PBS at 4 °C for 24 h, with the dialysis buffer being replaced every 6 h to ensure efficient removal of low-molecular-weight contaminants and residual medium components. To confirm MSCs, viable cells were analysed using flow cytometry. Flowcytometry was used in passage 4 to confirm Mesenchymal Stem Cells with positive CD 90, CD 105, CD 73 and negative CD 34, CD 45. Cells were cultivated in minimal essential medium (MEM) (Gibco BRL, Gaithersburg, MD, USA), Foetal Bovine serum (FBS) (Gibco BRL), and planted in a 100 mm tissue culture plate (Iwaki, Asahi, Japan) under normoxia conditions (CO_2_ 5%) and incubated at 37 °C. Mesenchymal Stem Cells (MSCs) are characterised not only by means of flowcytometry by confirming their identity via specific surface markers (positive for CD73, CD90, CD105; negative for haematopoietic markers such as CD45, CD34, CD14, CD19, and HLA-DR) but also demonstrating their ability for multilineage differentiation through staining, and assessing their distinct morphology (plastic-adherent, fibroblast-like) and proliferative capacity according to past research^24^, ^25^, ^26^.

qThe total protein concentration of the secretome was 2393.48 ng/mL (2.39 μg/mL)**,** which was used to standardize the administered dose across experimental animals. The secretome was then aliquoted and stored at −80 °C until use. Prior to in vivo administration, the secretome was thawed once and used immediately to avoid repeated freeze–thaw cycles. All secretome preparations were performed in triplicate to ensure experimental reproducibility.^13^^,^^27^

### 2.3 Nanoemulsion Propolis (NEP) preparation

Raw propolis sourced from stingless bees (*Tetragonula biroi*) was obtained from South Sulawesi, Indonesia, in March 2024, an area noted for normal temperatures of 25–27 °C and elevated humidity levels (about 84%). Two hundred 50 g of raw propolis underwent maceration with 96% ethanol as the extraction solvent for seven days at ambient temperature, with intermittent stirring. Ethanol was chosen for its superior efficacy in solubilizing polyphenols, flavonoids, and caffeic acid derivatives, which are the primary bioactive components of propolis. The extract was filtered and concentrated under reduced pressure at 40–50 °C utilizing a rotary evaporator (Büchi® R-20, Merck, Sigma-Aldrich, China) to get a viscous ethanolic extract. The concentrated extract was re-dissolved in 96% ethanol to create a 100% stock solution, which was then diluted with dimethyl sulfoxide (DMSO) and phosphate-buffered saline (PBS) to generate a series of working concentrations (100%–0.78%). The optical clarity of the propolis solution was assessed at 650 nm with a UV–Vis spectrophotometer (Shimadzu UV-2600i/2700i, Kyoto, Japan), and formulations exhibiting transmittance below 80% were subsequently studied using Triplot software. The creation of nanoemulsion was accomplished by combining ethanolic propolis extract with polysorbate as a surfactant and sodium tripolyphosphate as a stabilizing agent, thereafter subjected to high-speed homogenization at 4000 rpm for 60 min at ambient temperature. The nanoemulsion was created in three iterations to guarantee formulation consistency. The optimal effective concentration was determined to be 1.56% based on initial optimization and prior dose–response assessments. Consequently, a volume of 100 μL of NEP at this concentration was designated for administration across all treatment groups.^15^

### 2.4 Animal handling

The study used 35 male Wistar rats, each weighing around 200-250 g and aged 8-10 weeks. Animals were supplied from a laboratory animal center (Universitas Airlangga) and maintained under typical laboratory settings, including a 12-h light/dark cycle, a temperature of 22 ± 2 °C, and a relative humidity of 50-60%. Food and water were freely available. Before the trial, all rats were subjected to a one-week acclimatization phase to decrease stress and maintain physiological stability. At the end of the experimental period, the rats were deeply anesthetized by intraperitoneal administration of ketamine (100 mg/kg) combined with xylazine (10 mg/kg). After confirmation of deep anesthesia, the animals were euthanized by cervical dislocation, and the calvarial tissues were immediately harvested for further analysis. The investigation was approved by the Medical Research Ethics Committee of the Faculty of Mathematics and Natural Sciences at Universitas Sumatera Utara, under approval document number 0222/KEPH-FMIPA/2024.

### 2.5 Study design

The animals were randomly divided into seven experimental groups (n = 5 per group) as follows: K1: control group (no treatment); K2: 100 μL LPS only; K3: hyperglycemia (>230 mg/dL after streptozotocin induction); K4: 100 μL LPS + hyperglycemia; K5: LPS + hyperglycemia +100 μL nanoemulsion NEP (NEP); K6: LPS + hyperglycemia +100 μL HUC-MSC secretome; K7: LPS + hyperglycemia + combination of 100 μL HUC-MSC secretome and NEP. All treatments were administered once daily for seven consecutive days via intraperitoneal injection. Phosphate-buffered saline (PBS) was used as the vehicle in relevant groups. Prior to treatment, fasting blood glucose was measured from tail vein blood using a glucometer (Accu-Chek, Roche Diagnostics, Germany), and animals with glucose levels exceeding 230 mg/dL were considered hyperglycemic and included in the study. All samples were euthanized on day 8 using anesthesia and cervical dissection. Blood samples from each group were obtained and processed to obtain plasma for subsequent examination via ELISA. The calvarial tissue from each sample in each group was extracted, rinsed with PBS, and prepared for immunohistochemistry examination.

### 2.6 Enzyme-linked immunosorbent assay (ELISA)

Rat-specific ELISA kits were acquired from ThermoFisher Scientific (Massachusetts, USA) and utilized in accordance with the manufacturer's guidelines. Blood samples were obtained from the retro-orbital vein utilizing microcentrifuge tubes devoid of anticoagulant and permitted to coagulate at ambient temperature. The samples were subsequently centrifuged at 3000 rpm for 15 min at 4 °C to isolate serum, which was preserved at −80 °C until analysis. Capture antibodies were pre-coated onto microplate wells, and 100 μL of serum or standard solution was subsequently added to each well for the test. Following a 90–120 min incubation at room temperature, the wells were subjected to three washes with wash buffer to eliminate unbound components. An HRP-conjugated detection antibody was subsequently introduced and incubated for an additional 60 min. Subsequent to an additional washing step, the chromogenic reaction commenced with the addition of 3,3′,5,5′-tetramethylbenzidine (TMB) substrate. The reaction was halted with a sulfuric acid-based stop solution, and absorbance was recorded at 450 nm using a microplate reader. Cytokine concentrations were determined in pg/mL using the relevant standard curves.

### 2.7 Immunohistochemistry

Calvarial bone tissues were carefully excised from the rats and immediately fixed in 10% neutral buffered formalin for 24–48 h at room temperature (approximately 27 °C) to preserve tissue architecture and antigenicity. Following fixation, the tissues were subjected to a graded dehydration process through sequential immersion in increasing concentrations of ethanol to remove water content. This was followed by clearing in xylene to remove residual alcohol and facilitate paraffin infiltration. The tissues were then embedded in molten paraffin wax to obtain tissue blocks suitable for sectioning.

Using a rotary microtome, tissue sections of 4–5 μm thickness were cut and mounted onto poly-L-lysine–coated glass slides to enhance tissue adhesion. The slides were incubated at 60 °C for at least 1 h to ensure proper adherence and to melt residual paraffin. After deparaffinization in xylene, the sections were rehydrated through decreasing concentrations of ethanol until distilled water was reached. For antigen retrieval, the slides were immersed in citrate buffer solution and heated in a water bath or microwave at 95–100 °C for 15–20 min, followed by cooling to room temperature and rinsing in phosphate-buffered saline (PBS). To block endogenous peroxidase activity and minimize background staining, the sections were treated with 3% hydrogen peroxide in methanol for 10 min. Non-specific binding sites were blocked by incubation with normal goat serum or 1–5% bovine serum albumin (BSA) for 15–30 min at room temperature. The sections were then incubated overnight at 4 °C with primary antibodies specific for NF-κB, HSP-10, HSP-70, and IL-6, diluted according to the manufacturers’ instructions. After rinsing with PBS to remove unbound antibodies, the slides were incubated with horseradish peroxidase (HRP)-conjugated secondary antibodies for 30–60 min at room temperature. Immunoreactivity was visualized using 3,3′-diaminobenzidine (DAB) as the chromogenic substrate, producing a brown precipitate at the sites of antigen localization. The reaction was terminated by rinsing in distilled water. The sections were counterstained with hematoxylin, dehydrated through graded alcohols, cleared in xylene, and mounted with a permanent mounting medium. Microscopic examination was performed using a light microscope. The expression of target proteins was evaluated semi-quantitatively by assessing staining intensity and by counting the number of positively stained cells per microscopic field. The macrophages in the affected calvaria tissue were analysed by two observers blindly in five separate areas under a microscope at 100x, 400x, and 1000× magnification. The macrophages were recognized by brown precipitate and showed positive expression of NF-κB, IL-6, HSP-70, and HSP-10.

### 2.8 Statistical analysis

The statistical analysis utilized SPSS version 20.0, developed by IBM Corporation, located in Chicago, Illinois, USA. Quantitative data were presented as mean ± standard deviation (SD). Differences between groups for normally distributed (parametric) data were analysed using one-way analysis of variance (ANOVA), with subsequent application of Tukey's post hoc test. The Kruskal–Wallis test was utilized for non-parametric data analysis. A p-value below 0.05 is regarded as statistically significant.

## 3 Results

### 3.1 Modulation of cytokine expression in serum by HUC-MSC secretome and NEP in LPS-induced osteolysis under hyperglycemic conditions

**Fig. 1:**
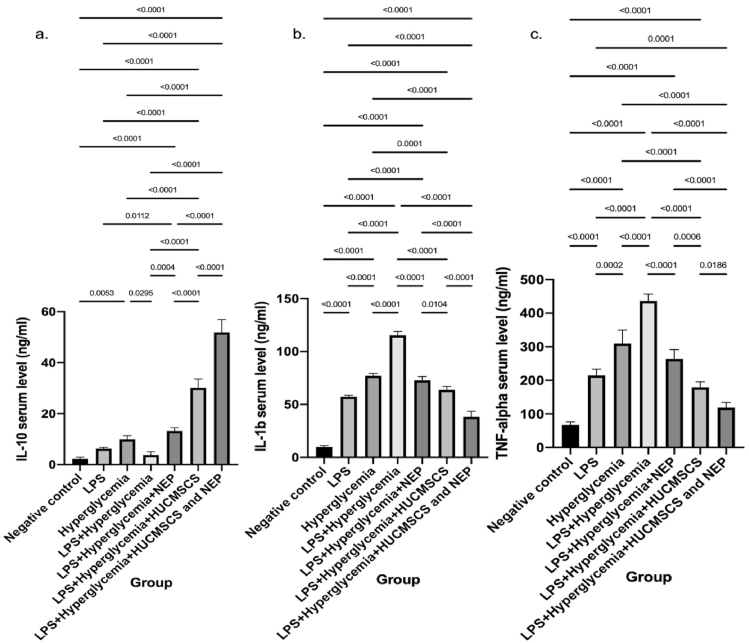
Serum cytokine levels in Wistar rats across treatment groups. (a) Interleukin-10 (IL-10), (b) Interleukin-1β (IL-1β), and (c) Tumor Necrosis Factor-α (TNF-α) were quantified by ELISA. Rats exposed to LPS and hyperglycemia (K4) exhibited significantly elevated IL-1β and TNF-α and reduced IL-10 compared to the control group (K1) and single-treatment groups (K2, K3). Administration of nanoemulsion propolis (NEP; K5) or HUC-MSC secretome (K6) partially normalized cytokine expression, while the combination therapy (K7) restored IL-10 and markedly suppressed IL-1β and TNF-α levels. Data are expressed as mean ± SD (n = 5 per group). Statistical significance: p < 0.05; p < 0.01; p < 0.001; p < 0.0001.

The ELISA test results for cytokine levels in mice serum demonstrated an expression pattern consistent with the treatment's inflammatory activity and immune modulation effects. The group administered LPS and exhibiting hyperglycemia (K4) demonstrated a significantly elevated level of IL-1β and TNF-α, two key pro-inflammatory cytokines associated with osteolysis, compared to the negative control group (K1), LPS alone (K2), or hyperglycemia alone (K3). This indicates that the interaction between LPS and hyperglycemia intensifies the systemic inflammatory state. Treatment with NEP (K5), HUC-MSC secretome (K6), or their combination (K7) significantly reduced levels of IL-1β and TNF-α (Fig. 1). The most notable decrease transpired in group K7, indicating a synergistic interaction between the two therapeutic agents. In contrast, the levels of IL-10, an anti-inflammatory cytokine, were negligible in the inflammation group (K4) and significantly elevated in the treatment group, reaching a high in K7. The results indicate that the combination of NEP and HUC-MSC secretome not only reduces the inflammatory response but also significantly enhances the anti-inflammatory immune response. Statistically, almost all group comparisons demonstrated significant changes (p < 0.05 to p < 0.0001), substantiating the notion that this combination therapy efficiently regulates the production of cytokines implicated in the pathophysiology of LPS-induced osteolysis and hyperglycemia.

### 3.2 NF-κB expression in calvarial tissue by HUC-MSC secretome and NEP on LPS-induced osteolysis under hyperglycemic conditions

**Fig. 2:**
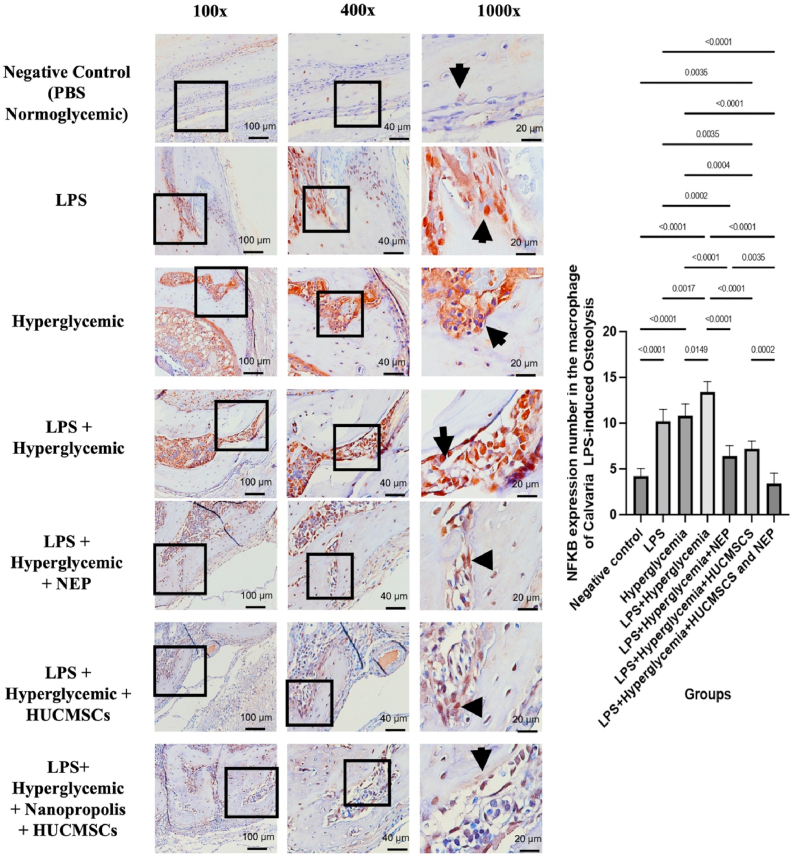
NF-κB expression in calvarial macrophages of Wistar rats across treatment groups. Representative images at magnifications 100 × , 400 × , and 1000 × show NF-κB–positive cells (brown nuclear staining, indicated by arrows) in the calvarial bone tissue. The negative control group (K1) received no treatment; K2 received LPS; K3 received hyperglycemia induction; K4 received LPS and hyperglycemia; K5 received LPS, hyperglycemia, and 100 μL NEP; K6 received LPS, hyperglycemia, and 100 μL HUC-MSC; and K7 received LPS, hyperglycemia, and combination treatment of HUC-MSC and NEP. Statistical significance: p < 0.05; p < 0.01; p < 0.001; p < 0.0001. (For interpretation of the references to colour in this figure legend, the reader is referred to the Web version of this article.)

A significant disparity in NF-κB levels exists among the therapy groups. In the negative control group (normoglycemic PBS), NF-κB expression was very low, as indicated by the minimum positive staining detected in the macrophage cell nucleus. In contrast, the groups treated with LPS (K2) and those experiencing hyperglycemia (K3) demonstrated increased NF-κB expression, notably more prominent in the combined group of LPS and hyperglycemia (K4). In this group, macrophage cells exhibited significant activation, as indicated by the pronounced brown staining in the cell nucleus. This suggests that the interaction between systemic inflammatory stimuli and metabolic stress can collaboratively activate the NF-κB pathway in cranial tissue. The administration of NEP treatment (K5) and HUC-MSC secretome (K6) separately led to a decrease in NF-κB expression relative to the inflammatory group. The most significant reduction was observed in the cohort receiving both treatments (K7), with expression levels approaching those of the control group. The quantitative analysis corroborated these findings, demonstrating significant disparities across the groups (p < 0.05 to p < 0.0001), with K4 displaying the highest quantity of NF-κB positive macrophage cells, whilst K1 and K7 presented the lowest levels (Fig. 2). The findings support the notion that the concurrent application of HUC-MSC and NEP therapy produces a synergistic anti-inflammatory effect, effectively suppressing macrophage activation and NF-κB expression in LPS-induced osteolysis under hyperglycemic circumstances.

### 3.3 HSP-70 expression in calvarial tissue by HUC-MSC secretome and NEP on LPS-induced osteolysis under hyperglycemic conditions

**Fig. 3:**
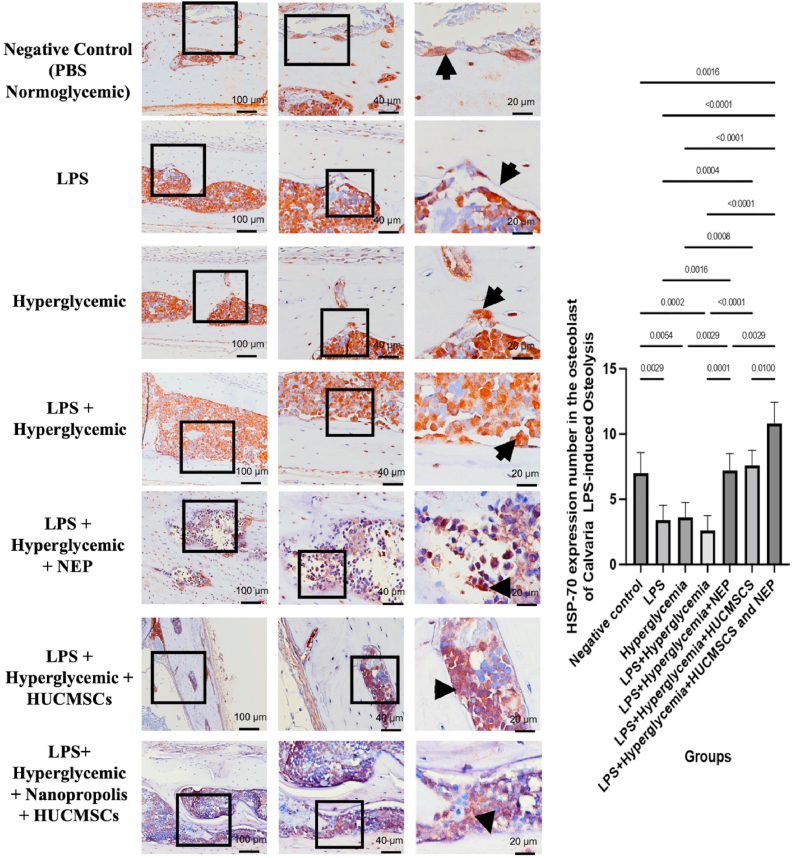
HSP-70 expression in calvarial macrophages of Wistar rats across treatment groups. Representative images at magnifications 100 × , 400 × , and 1000 × show HSP–70 positive cells (brown nuclear staining, indicated by arrows) in the calvarial bone tissue. The negative control group (K1) received no treatment; K2 received LPS; K3 received hyperglycemia induction; K4 received LPS and hyperglycemia; K5 received LPS, hyperglycemia, and 100 μL NEP; K6 received LPS, hyperglycemia, and 100 μL HUC-MSC; and K7 received LPS, hyperglycemia, and combination treatment of HUC-MSC and NEP. Statistical significance: p < 0.05; p < 0.01; p < 0.001; p < 0.0001. (For interpretation of the references to colour in this figure legend, the reader is referred to the Web version of this article.)

In the negative control group (normoglycemic PBS), HSP-70 expression was significantly increased, as seen by brown staining in the osteoblast cytoplasm, reflecting typical homeostatic conditions. In contrast, both the LPS and hyperglycemia groups demonstrated a significant decrease in HSP-70 expression, indicating inflammatory and metabolic stress that compromises cellular defensive mechanisms. The lowest HSP-70 expression was seen in the LPS + hyperglycemia group (K4), signifying considerable osteolysis and osteoblast dysfunction. The injection of NEP (K5) and HUC-MSC secretome (K6) led to an increase in HSP-70 expression in osteoblasts, although not to the level seen in the control group. In the HUC-MSC + NEP combination group (K7), HSP-70 expression significantly elevated, approaching physiological levels, as evidenced by the more extensive and intense positive staining. The bar graph on the right supports the histology findings, demonstrating statistically significant differences (p < 0.05 to p < 0.0001) among the groups (Fig. 3). The data suggest that combination therapy can restore HSP-70 expression as a protective mechanism for osteoblasts against inflammatory and hyperglycemic stress that causes osteolysis. These findings underscore the collaborative role of MSC secretome and NEP in promoting bone cell regeneration by elevating the concentrations of protective proteins, including HSP-70.

### 3.4 HSP-10 expression in calvarial tissue by HUC-MSC secretome and NEP on LPS-induced osteolysis under hyperglycemic conditions

**Fig. 4:**
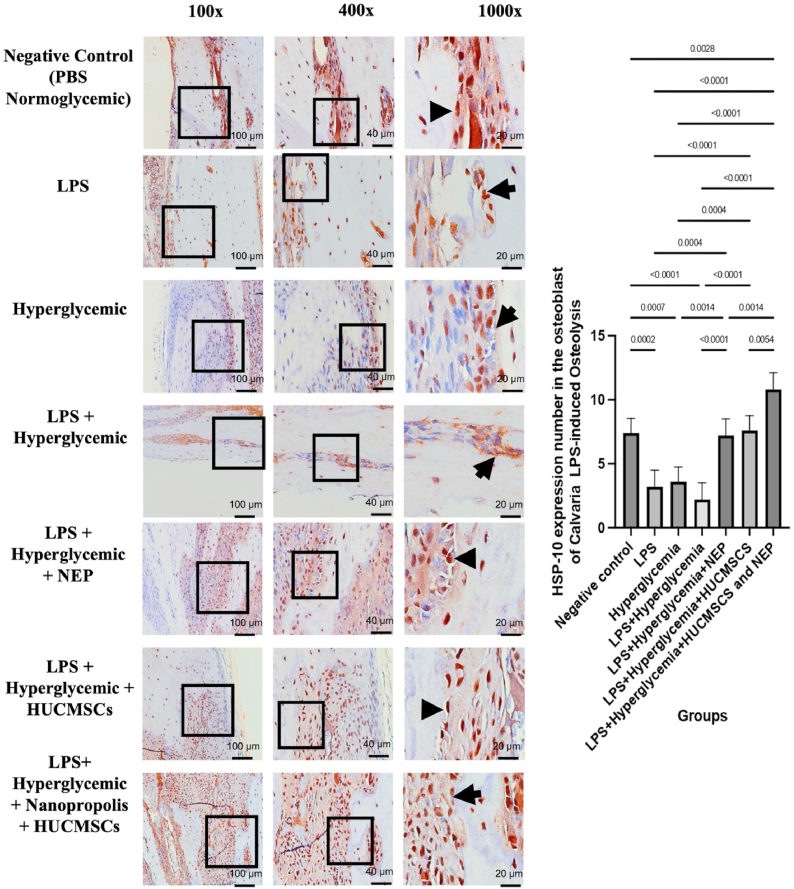
HSP-10 expression in calvarial macrophages of Wistar rats across treatment groups. Representative images at magnifications 100 × , 400 × , and 1000 × show HSP–10 positive cells (brown nuclear staining, indicated by arrows) in the calvarial bone tissue. The negative control group (K1) received no treatment; K2 received LPS; K3 received hyperglycemia induction; K4 received LPS and hyperglycemia; K5 received LPS, hyperglycemia, and 100 μL NEP; K6 received LPS, hyperglycemia, and 100 μL HUC-MSC; and K7 received LPS, hyperglycemia, and combination treatment of HUC-MSC and NEP. Statistical significance: p < 0.05; p < 0.01; p < 0.001; p < 0.0001. (For interpretation of the references to colour in this figure legend, the reader is referred to the Web version of this article.)

The stable physiological condition of bone tissue resulted in the negative control group (normoglycemic PBS) displaying a comparatively elevated level of HSP-10 expression. Conversely, the reduced intensity of brown staining in osteoblasts indicated a significant decrease in HSP-10 expression in the LPS, hyperglycemia, and especially the LPS + hyperglycemia groups. This offers more evidence that the simultaneous decrease of HSP-10 expression due to inflammation and metabolic stress undermines cellular defense mechanisms, harms osteoblasts, and exacerbates osteolysis. A partial restoration of HSP-10 expression was evidenced by an increase in the quantity of osteoblast cells exhibiting positive staining following the administration of NEP and HUC-MSC secretome independently. The group administered both NEP and HUC-MSC had the most significant effect, with HSP-10 expression aligning with that of the control group. This evidence suggests the potential for a synergistic effect of the two therapies in reactivating cellular protective mechanisms through the upregulation of HSP-10 expression. Significant differences exist among the groups (p < 0.05 to p < 0.0001), with the combination therapy group exhibiting the highest quantity of HSP-10-positive osteoblasts, as illustrated in the right-hand bar graph. Fig. 4 illustrates that stem cell secretome-based therapy and NEP enhance cellular protective mechanisms in bone by stimulating chaperone proteins such as HSP-10, hence reinforcing the notion that both interventions mitigate inflammatory pathways.

### 3.5 IL-6 expression in calvarial tissue by HUC-MSC secretome and NEP on LPS-induced osteolysis under hyperglycemic conditions

**Fig. 5:**
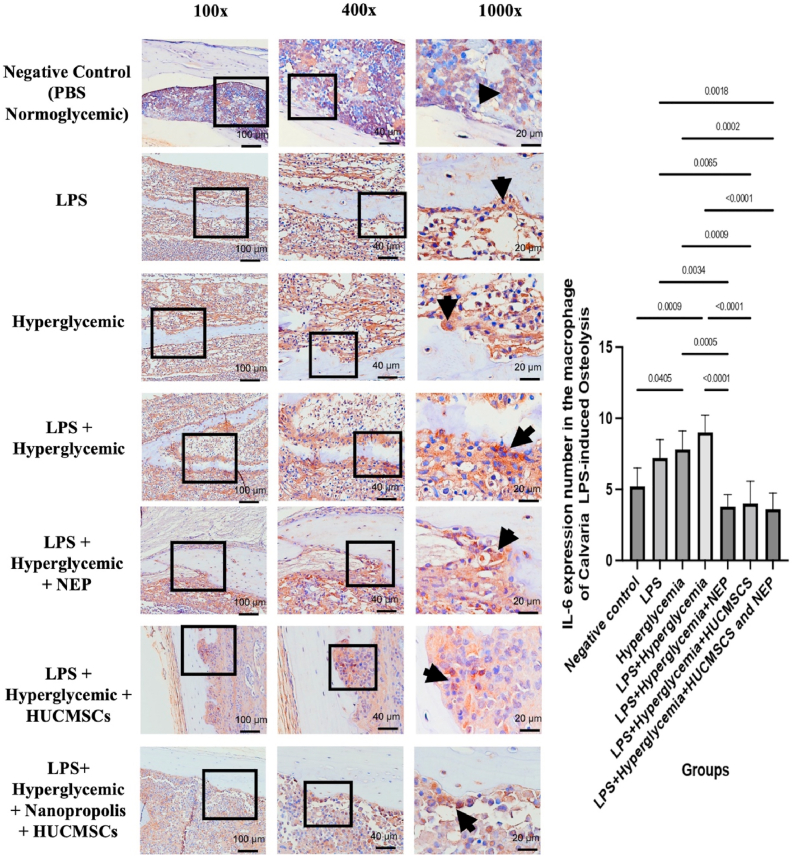
IL-6 expression in calvarial macrophages of Wistar rats across treatment groups. Representative images at magnifications 100 × , 400 × , and 1000 × show IL-6 positive cells (brown nuclear staining, indicated by arrows) in the calvarial bone tissue. The negative control group (K1) received no treatment; K2 received LPS; K3 received hyperglycemia induction; K4 received LPS and hyperglycemia; K5 received LPS, hyperglycemia, and 100 μL NEP; K6 received LPS, hyperglycemia, and 100 μL HUC-MSC; and K7 received LPS, hyperglycemia, and combination treatment of HUC-MSC and NEP. Statistical significance: p < 0.05; p < 0.01; p < 0.001; p < 0.0001. (For interpretation of the references to colour in this figure legend, the reader is referred to the Web version of this article.)

Fig. 5 illustrates that the negative control group (normoglycemic PBS) exhibits minimal IL-6 expression, indicating a stable tissue condition devoid of inflammatory activation. The LPS, hyperglycemia, and particularly the LPS + hyperglycemia (K4) groups exhibited a notable increase in IL-6 expression, indicated by the pronounced brown staining intensity in the cytoplasm of macrophages (denoted by arrows). This suggests that systemic inflammation combined with metabolic stress synergistically enhances IL-6 expression, exacerbating the local inflammatory response and facilitating osteoclastogenesis.

The administration of NEP therapy (K5) or HUC-MSC secretome (K6) individually resulted in a reduction of IL-6 expression when compared to the LPS + hyperglycemia group, demonstrating the anti-inflammatory properties of each treatment. The combination group (K7: NEP + HUC-MSC) exhibited the most notable effect, demonstrating a significant reduction in IL-6 expression, nearly aligning with the negative control group. This reduction suggests a synergistic effect in inhibiting the expression of proinflammatory cytokines, which are primary targets in the management of inflammatory osteolysis. The quantitative bar graph on the right supports the histological findings, with statistical analysis indicating significant differences between groups (p < 0.05 to p < 0.0001). The data indicate that the combination intervention effectively inhibits macrophage activation and reduces IL-6 production, thereby decreasing the risk of bone tissue destruction associated with chronic inflammation.

**Fig. 6:**
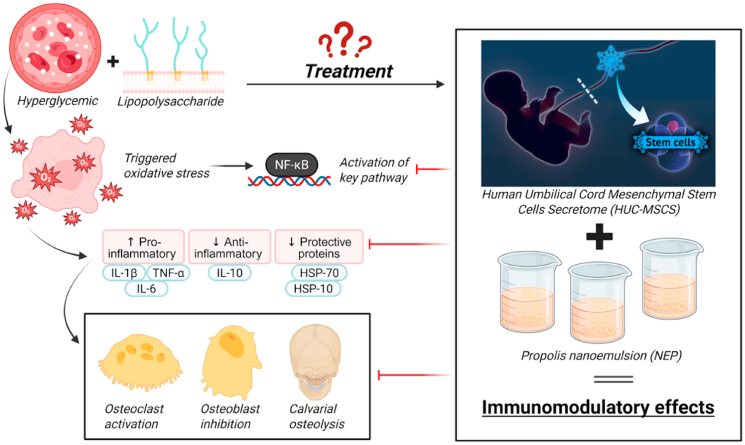
Graphical summary of the study. Hyperglycemia and LPS induce NF-κB activation, leading to increased pro-inflammatory cytokines, reduced IL-10, and decreased HSP-70/HSP-10, which drive osteoclast activation and calvarial osteolysis. Treatment with HUC-MSC secretome and NEP (NEP) modulates these pathways, restoring protective balance and exerting immunomodulatory effects (Created with BioRender.com).

The summarize the expression of inflammatory, anti-inflammatory, stress-related, and signaling molecular markers across all experimental groups, including descriptive statistics (mean ± SD), normality and homogeneity test results, and between-group comparisons, demonstrating statistically significant differences where applicable (p < 0.05) in supplementarry file (Tables S1 and S2). The results of this study demonstrate that under hyperglycemic conditions combined with lipopolysaccharide (LPS) exposure, oxidative stress is triggered, leading to activation of the NF-κB pathway. This activation caused a significant increase in pro-inflammatory cytokines (IL-1β, TNF-α, and IL-6), accompanied by reduced anti-inflammatory signaling (IL-10) and suppression of protective stress proteins (HSP-70 and HSP-10).^4^^,^^28^ These molecular changes were associated with enhanced osteoclast activation, inhibition of osteoblast activity, and subsequent calvarial osteolysis. In contrast, treatment with human umbilical cord mesenchymal stem cell secretome (HUC-MSC) in combination with NEP effectively modulated these pathological processes. The combined therapy downregulated NF-κB activation and pro-inflammatory cytokines, restored IL-10 expression, and reactivated stress-protective proteins, thereby attenuating osteoclastogenesis and preserving osteoblast viability. Collectively, these immunomodulatory effects explain the observed reduction in calvarial osteolysis in the treated groups (Fig. 6).

## 4 Discussion

Osteolysis in systemic inflammatory disorders, especially those exacerbated by hyperglycemia, arises from a dysregulation of osteoclast and osteoblast activity, mediated by the cytokine network.^29^ The calvarial bone was chosen as the experimental location for osteolysis assessment due to its status as a non-weight-bearing flat bone characterized by elevated metabolic activity and heightened sensitivity to inflammatory stimuli. In contrast to long bones, which are significantly affected by mechanical loading and systemic biomechanical factors, the calvarial bone model facilitates direct and repeatable evaluation of inflammation-induced bone resorption. Additionally, LPS-induced calvarial osteolysis serves as a recognized and extensively utilized experimental model for investigating inflammatory bone degradation and treatment strategies under metabolic stress, such as hyperglycemic circumstances. Consequently, the utilization of calvarial bone in this investigation was deemed the most suitable model for the targeted examination of inflammation-mediated osteolysis. Increased levels of proinflammatory cytokines, such as IL-1β and TNF-α, promote osteoclast development and activation through the NF-κB, RANK/RANKL, and MAPK signaling pathways, leading to the destruction of the bone matrix.^30^ The rat model that was exposed to both LPS and hyperglycemia showed a significant increase in cytokine levels, which suggests a chronic inflammatory condition that promotes the gradual loss of bone. Previous studies have shown that LPS causes macrophages to release IL-1β and TNF-α, and that chronic hyperglycemia, through the creation of AGEs and oxidative stress, worsens the inflammatory response.^6^^,^^31^ The administration of HUC-MSC significantly suppressed the expression of IL-1β and TNF-α.^31^ The observed effects can be attributed to the bioactive components present in the secretome, including TGF-β, IL-10, microRNA, and exosomes, which are recognized for their role in inhibiting M1 macrophage activation and diminishing inflammatory gene expression.^32^ Through their secretome, MSCs encourage macrophage polarization toward the M2 phenotype, which is associated with anti-inflammatory and reparative roles, and it enhances osteoblast activity.^33^ Simultaneously, NEP exhibits a notable immunomodulatory role. The concentrations of flavonoids and phenolic compounds in NEP, particularly CAPE (Caffeic Acid Phenethyl Ester), have demonstrated the ability to block NF-κB activation and obstruct the transcriptional synthesis of proinflammatory cytokines.^34^ The nanoemulsion formulation improves the bioavailability of NEP, expedites systemic absorption, and facilitates its distribution to target tissues, such as bone.^34^ The combination of HUC-MSC and NEP demonstrated the most significant reduction in IL-1β and TNF-α levels, while concurrently elevating IL-10 levels. This indicates that the two drugs operate synergistically, yielding a more substantial combined benefit than either therapy individually. IL-10 plays a crucial role in alleviating inflammation by obstructing the synthesis of proinflammatory cytokines and dampening inflammatory signaling pathways, in addition to facilitating tissue regeneration. Thus, increased IL-10 levels in the combo group indicate the effectiveness of immunological regulation.^17^ The decision to integrate HUCMSC secretome and NEP was predicated on their synergistic biological effects. The secretome provides immunomodulatory and regenerative factors, while NEP has significant antioxidant and NF-κB inhibitory properties. This combined approach offers a more extensive preventive effect than each medication individually, especially in the hyperglycemic context where oxidative stress and inflammation are pivotal in bone resorption. This synergy validates the comprehensive therapeutic approach and differentiates it from monotherapy therapies.

Immunohistochemistry findings on rat calvarial tissue demonstrated a notable elevation in NF-κB activation under osteolytic circumstances generated by a combination of lipopolysaccharide (LPS) and hyperglycemia. NF-κB functions as a crucial transcription factor that governs the production of various proinflammatory genes, including TNF-α, IL-1β, and IL-6. The activation of this pathway in macrophages is crucial for bone inflammation, largely via facilitating osteoclastogenesis and suppressing osteoblast activity.^35^ The group administered LPS and exhibiting hyperglycemia showed elevated NF-κB expression, suggesting that this combination of stimuli fostered a significant proinflammatory environment and contributed to bone tissue damage.^36^ In comparison to the group treated with LPS and hyperglycemia, NF-κB expression was significantly reduced following administration of either NEP or HUC-MSC secretome alone.^18^ The combination of HUC-MSC secretome and NEP resulted in a notable reduction in NF-κB expression, approaching the levels observed in the negative control group. The findings indicate a synergistic effect between the two therapies, which complementarily inhibit inflammatory pathways. This combination therapy suggests potential for modulating the bone microenvironment affected by systemic inflammation and metabolic stress.

The immunohistochemistry research indicates that HSP-70 expression in osteoblasts of calvarial tissue is crucial for sustaining bone cell homeostasis during inflammatory and metabolic stress situations. HSP-70 is a chaperone protein that is activated in situations of cellular stress, such as inflammation, hyperglycemia, and tissue injury.^28^^,^^37^ It plays a critical role in preventing protein aggregation, facilitating cell repair, and inhibiting apoptosis.^38^^,^^39^ A notable reduction in HSP-70 expression in the group subjected to LPS and hyperglycemia suggests that these conditions foster a pathological environment that impairs the protective role of osteoblast cells, exacerbates the osteolysis process, and diminishes the capacity of bone tissue to preserve its structural integrity.^40^ The administration of a single therapy, either as NEP or HUC-MSC, has demonstrated an ability to enhance HSP-70 expression, although it has not attained the physiological expression level observed in the control group. This suggests that both agents can alleviate cellular stress and activate intrinsic protective mechanisms. NEP, containing bioactive compounds like CAPE and flavonoids, exhibits antioxidant and anti-inflammatory properties that enhance HSP-70 expression.^34^^,^^41^ The HUC-MSC secretome consists of several growth factors and exosomes that stimulate regeneration pathways and enhance the expression of cytoprotective proteins, including HSP-70.

The expression of HSP-10 (Heat Shock Protein 10) in rat calvarial tissue exhibits notable variations in cellular responses to inflammatory and metabolic stress induced by LPS and hyperglycemia. HSP-10 is a mitochondrial chaperone protein that facilitates correct protein folding, safeguards cells from oxidative damage, and sustains metabolic stability during stress circumstances.^42^ HSP-10 plays a crucial role in preserving osteoblast viability and function within the bone, particularly under conditions of chronic inflammation that may induce osteolysis.^28^ A significant decrease in HSP-10 expression was noted in the LPS, hyperglycemia, and especially in the combined LPS + hyperglycemia groups, indicating that these variables collaboratively impede cellular defensive mechanisms in osteoblasts. This syndrome impairs the capacity of osteoblasts to maintain bone matrix homeostasis, resulting in elevated osteoclast activity and augmented bone resorption.^43^ The results support previous studies demonstrating that systemic inflammation and chronic hyperglycemia result in mitochondrial dysfunction and prolonged oxidative stress, thus reducing the expression of protective proteins such as HSP-10. The individual application of NEP and secretome HUC-MSC treatment led to a partial restoration of HSP-10 expression. This impact is likely due to the strong antioxidant characteristics of NEP and the bioactive constituents in the MSC secretome, such as growth factors and exosomes, which can improve mitochondrial function and stimulate the development of protective proteins. The cohort administered the combination of NEP and HUC-MSC demonstrated the most pronounced elevation in HSP-10 expression, approaching physiological values.^17^ Based on our previous analysis, the (NEP) of *T. biroi* demonstrated positive phytochemical reactions for alkaloids, flavonoids, and phenols. Characterization using Particle Size Analyzer (PSA) confirmed that the NEP exhibited a particle size range of 151.28–182.2 nm, which falls within the acceptable nanoparticle range (10–1000 nm). In addition, the zeta potential value was 32.76 mV, indicating good colloidal stability (normal value ± 30 mV) and supporting the ability of the formulation to penetrate tissue barriers effectively. This physicochemical profile elucidates the molecular basis for the augmented biological activity of the NEP employed in our research. The HUCMSC secretome constitutes a complex amalgamation of soluble biomolecules that are actively released during cellular proliferation. Prior research indicate that it encompasses a diverse array of cytokines and growth factors, including as IL-10 and TGF-β, which are crucial in regulating inflammation. Furthermore, angiogenic mediators, including VEGF and hepatocyte growth factor (HGF), are present, facilitating vascularization and the survival of osteoblasts. In addition to these proteins, the secretome contains extracellular vesicles and exosomes that are abundant with regulatory microRNAs, lipids, and peptides, which affect immune cell polarization and tissue regeneration. The bioactive components collectively confer immunomodulatory, anti-inflammatory, and regenerative properties to the secretome, rendering it a potentially acellular treatment option for inflammatory bone illnesses.

Interleukin-6 (IL-6) is a proinflammatory cytokine that is central to the pathogenesis of numerous inflammatory diseases, such as osteolysis.^44^^,^^45^ In bone tissue, IL-6 serves as a crucial mediator in osteoclast differentiation and activation, enhancing the inflammatory pathway via interactions with macrophages and other immune cells.^30^^,^^46^ The immunohistochemistry results indicated a significant increase in IL-6 expression in the calvarial tissue of rats subjected to LPS exposure and hyperglycemia.^47^ This discovery indicates that inflammation and metabolic stress collaboratively augment macrophage activation and the generation of proinflammatory cytokines. The marked increase in IL-6 levels in the LPS + hyperglycemia group suggests that the interaction between bacterial endotoxin and persistent hyperglycemia creates a milieu conducive to bone inflammation and matrix resorption. This corresponds with previous studies demonstrating that IL-6 increases RANKL expression, facilitates osteoclastogenesis, and results in structural bone deterioration in chronic inflammatory states.^48^ The administration of NEP and HUC-MSC therapy, whether administered individually or in combination, significantly reduced IL-6 expression. NEP comprises anti-inflammatory compounds, including flavonoids and CAPE, which are recognized for their ability to inhibit the transcription of inflammatory genes such as IL-6 via the suppression of the NF-κB pathway.^41^ A range of bioactive chemicals, such as IL-10, TGF-β, and exosomes, are found in the HUC-MSC secretome. These molecules help control the immune response, inhibit M1 macrophages, and encourage the resolution of inflammation.^27^ The cohort administered the combination of both therapies exhibited the most substantial reduction in IL-6 expression, nearly matching that of the negative control group. The results demonstrate a synergistic effect in inhibiting the macrophage inflammatory response and IL-6 production, potentially serving as a primary mechanism for preventing additional bone damage.^17^^,^^49^

**Fig. 7:**
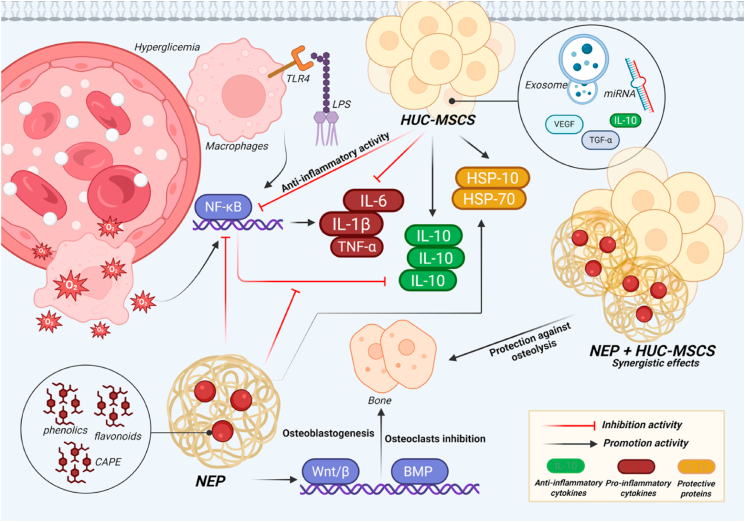
Synergistic mechanism of HUC-MSC secretome and NEP in hyperglycemia and LPS-induced osteolysis. The therapy suppresses NF-κB–mediated pro-inflammatory cytokines, restores IL-10 and HSPs, and promotes osteoblastogenesis, thereby protecting against bone resorption (Created with BioRender.com).

Fig. 7 illustrates the molecular route that highlights the synergistic interaction between HUC-MSC secretome and NEP in alleviating hyperglycemia- and LPS-induced calvarial osteolysis. Hyperglycemia and LPS stimulate TLR4 signaling in macrophages, leading to increased oxidative stress and NF-κB activation, which enhances the release of pro-inflammatory cytokines including IL-1β, TNF-α, and IL-6.^50^^,^^51^ The inflammatory cascade, along with diminished IL-10 expression and inhibition of protective proteins (HSP-70 and HSP-10), facilitates osteoclastogenesis and bone resorption while hindering osteoblast function. The administration of HUC-MSC secretome delivers immunomodulatory and regenerative agents, including as IL-10, TGF-β, VEGF, and exosome-associated miRNAs, which suppress NF-κB activation and reestablish anti-inflammatory equilibrium. Simultaneously, NEP provides phenolic compounds, flavonoids, and CAPE, which exhibit significant antioxidant and NF-κB inhibitory properties, while also facilitating osteoblastogenesis via Wnt/β-catenin and BMP signaling pathways. The integrated therapy consequently inhibits pro-inflammatory mediators, elevates IL-10 expression, reactivates HSP-70 and HSP-10, and reinstates the equilibrium between osteoclast and osteoblast activity. These actions collectively offer robust protection against osteolysis in hyperglycemic circumstances, highlighting the therapeutic synergy between HUC-MSC secretome and NEP. Compared with our previous work,^24^ which mainly demonstrated the suppression of osteoclastogenesis markers such as NFATc1, RANK/RANKL/OPG, sclerostin, TRAP, and cathepsin K, the present study provides extended mechanistic insights. Here, we show that the combination of HUC-MSC secretome and NEP not only attenuates osteoclastogenic signaling but also regulates inflammatory cytokines (IL-1β, TNF-α, IL-6, IL-10, NF-κB) and restores stress proteins (HSP-70 and HSP-10).^16^^,^^24^ This dual activity signifies that the medication has both anti-inflammatory and cytoprotective effects in hyperglycemic situations. These findings constitute a novel contribution by expanding the understanding of therapeutic mechanisms and underscoring the significance of this combination therapy for diabetic bone loss, a clinical condition previously unexamined in other investigations.

This study has several limitations that should be acknowledged. The evaluation of NF-κB, IL-6, HSP-70, and HSP-10 expression relied on semi-quantitative immunohistochemistry; therefore, future studies incorporating fully quantitative molecular analyses such as Western blotting or real-time PCR would strengthen mechanistic validation. The experimental duration was limited to a short-term observation period, and long-term effects of HUC-MSC secretome and NEP combination therapy on bone remodeling were not assessed. Despite these limitations, the present study provides robust mechanistic insights into inflammatory modulation and cytoprotective responses under hyperglycemic osteolysis conditions.

## 5 Conclusion

This research illustrates that the amalgamation of human umbilical cord mesenchymal stem cell (HUC-MSC) secretome and NEP produces a synergistic therapeutic impact on hyperglycemia-induced calvarial osteolysis. The combined therapy markedly reduced NF-κB activation, inhibited pro-inflammatory cytokines (IL-1β, TNF-α, and IL-6), increased the anti-inflammatory cytokine IL-10, and reinstated protective stress proteins (HSP-70 and HSP-10). These molecular modifications combined diminished osteoclast activation, maintained osteoblast viability, and safeguarded bone integrity under metabolic and inflammatory stress conditions. The results indicate that the combined effect of HUC-MSC secretome and NEP not only modulates inflammatory pathways but also restores cytoprotective mechanisms, presenting an innovative non-cellular, natural product-based approach for addressing diabetic bone loss. Additional investigations, encompassing extensive preclinical and translational trials, are necessary to confirm the clinical efficacy of this combination medication in averting craniofacial and systemic bone problems linked to hyperglycemia.

## Patient's and Guardian's consent

“Not applicable.

This study did not involve human participants; therefore, patient and guardian consent was not required.”

## Study ethical clearance

The investigation was approved by the Medical Research Ethics Committee of the Faculty of Mathematics and Natural Sciences at Universitas Sumatera Utara, under approval document number 0222/KEPH-FMIPA/2024.

## Funding

This research was funded by the 10.13039/501100002385Ministry of Higher Education, Science, and Technology of the Republic of Indonesia under the Strategic Collaborative Research Scheme (KATALIS) 2024. The study was carried out in collaboration with Universitas Gadjah Mada (UGM), Universitas Negeri Malang (UM), Universitas Sumatera Utara (USU), and Universitas Airlangga (UNAIR). This project was supported under Grant Number DRTPM 047/E5/PG.02.00/PL.BATCH.2/2024 and Agreement/Contract Number 4/UN5.4.10.K/PPM/KP.DRTPM-KATALIS/2024.

## Declaration of competing interest

The authors declare that they have no known competing financial interests or personal relationships that could have appeared to influence the work reported in this paper.
