# Supplementary material for: HUC-MSC secretome and Nanoemulsion Propolis synergistically modulate inflammatory responses in hyperglycemia-induced calvarial osteolysis
Source: J Oral Biol Craniofac Res. 2026 Mar 2;16(2):101430. doi: 10.1016/j.jobcr.2026.101430 (PMC12968417; doi:10.1016/j.jobcr.2026.101430)
Supplement: Multimedia component 1 [file mmc1.docx]

**Table S1.** Expression of various molecular markers Mean ± Standar Deviation (SD), Homogenity and normality test result in each group.

| **Molecular Marker** | **Group (Mean ± Standar Deviation (SD))** | | | | | | | **Homogenity test p-value** |
| --- | --- | --- | --- | --- | --- | --- | --- | --- |
|  | **Negative Control** | **LPS** | **Hyperglycemia** | **LPS+**  **Hyperglycemia** | **LPS+**  **Hyperglycemia+NEP** | **LPS+**  **Hyperglycemia**  **+HUCMSCS** | **LPS+**  **Hyperglycemia**  **+HUCMSCS+**  **NEP** |  |
| IL-1β | 9.69 ± 1.314 | 57.34 ± 1.37 | 77.11± 2.26 | 115.3 ± 3.59 | 72.83 ± 3.47 | 63.82 ± 3.29 | 38.35 ± 5.24 | 0.99* |
| Normality Test  p-value | 0.53* | 0.85* | 0.44* | 0.18* | 0.78* | 0.83* | 0.44* |  |
| TNF-α | 67.4 ± 8.99 | 214.8 ± 18.79 | 309.7 ± 40.28 | 436.7 ± 20.12 | 264 ± 27.38 | 179.2 ± 16.35 | 118.8 ± 15.15 | 0.97* |
| Normality Test  p-value | 0.85* | 0.51* | 0.41* | 0.34* | 0.66* | 0.83* | 0.98* |  |
| IL-10 | 2.39 ± 0.52 | 6.29 ± 0.47 | 9.93 ± 1.45 | 3.76 ± 1.32 | 13.25 ± 1.29 | 30.13 ± 3.48 | 51.85 ± 5.05 | 0.98* |
| Normality Test  p-value | 0.82* | 0.91* | 0.60* | 0.98* | 0.99* | 0.99* | 0.4* |  |
| IL-6 | 8 ± 1.58 | 4 ± 1.14 | 3.2 ± 1.3 | 2.4 ± 1.14 | 7.4 ± 1.14 | 8.4 ± 1.14 | 11.2 ± 1.3 | 0.76* |
| Levene`s Normality Test  p-value | 0.42* | 0.42* | 0.42* | 0.15* | 0.31* | 0.97* | 042* |  |
| NF-κB | 4.2 ± 0.4 | 10.2 ± 1.3 | 10.8 ± 1.3 | 13.4 ± 1.14 | 6.4 ± 1.14 | 7.2 ± 0.84 | 3.4 ± 1.14 | 0.92* |
| Normality Test  p-value | 0.31* | 0.42* | 0.42* | 0.81* | 0.81* | 0.31* | 0.81* |  |
| HSP-70 | 7 ± 1.58 | 3.4 ± 1.14 | 3.6 ± 1.14 | 2.6 ± 1.14 | 7.2 ± 1.3 | 7.6 ± 1.14 | 10.8 ± 1.64 | 0.84* |
| Normality Test  p-value | 0.97* | 0.81* | 0.81* | 0.81* | 0.42* | 0.81* | 0.49* |  |
| HSP-10 | 7.4 ± 1.14 | 3.2 ± 1.3 | 3.6 ± 1.14 | 2.2 ± 1.3 | 7.2 ± 1.3 | 7.6 ± 1.14 | 10.8 ± 1.3 | 0.87* |
| Normality Test  p-value | 0.81* | 0.42* | 0.81* | 0.42* | 0.42* | 0.81* | 0.42* |  |

**Information:** Sample for each group in every variable was 5 samples. *normality and homogeneity data for each group at p>0.05.
