# Supplementary material for: HUC-MSC secretome and Nanoemulsion Propolis synergistically modulate inflammatory responses in hyperglycemia-induced calvarial osteolysis
Source: J Oral Biol Craniofac Res. 2026 Mar 2;16(2):101430. doi: 10.1016/j.jobcr.2026.101430 (PMC12968417; doi:10.1016/j.jobcr.2026.101430)
Supplement: Multimedia component 2 [file mmc2.docx]

**Table S2.** Comparison of expression of various molecular markers between the groups.

| **Group** | **P-value for “between groups” comparison** | | | | | |  |
| --- | --- | --- | --- | --- | --- | --- | --- |
|  | **Molecular Marker** | | | | | |  |
|  | **IL-1β** | **TNF-α** | **IL-10** | **IL-6** | **NF-κB** | **HSP-10** | **HSP-70** |
| Negative control vs. LPS | 0.001* | 0.001* | 0.33 | 0.194 | 0.001* | 0.001* | 0.003* |
| Negative control vs. Hyperglycemia | 0.001* | 0.001* | 0.001* | 0.041 | 0.001* | 0.001* | 0,005* |
| Negative control vs. LPS+Hyperglycemia | 0.001* | 0.001* | 0.985 | 0.001* | 0.001* | 0.001* | 0.001* |
| Negative control vs. LPS+Hyperglycemia+NEP | 0.001* | 0.001* | 0.001* | 0.585 | 0.056 | 0.99 | 0.99 |
| Negative control vs. LPS+Hyperglycemia+HUCMSCS | 0.001* | 0.001* | 0.001* | 0.739 | 0.004* | 0.99 | 0.99 |
| Negative control vs. LPS+ Hyperglycemic +HUCMSCS+NEP | 0.001* | 0.06 | 0.001* | 0.432 | 0.912 | 0.003* | 0.002* |
| LPS vs. Hyperglycemia | 0.001* | 0.001* | 0.408 | 0.988 | 0.977 | 0.99 | 0.99 |
| LPS vs. LPS+Hyperglycemia | 0.001* | 0.001* | 0.777 | 0.298 | 0.002* | 0.856 | 0.958 |
| LPS vs. LPS+Hyperglycemia+NEP | 0.001* | 0.079 | 0.011* | 0.003* | 0.001* | 0.001* | 0.002* |
| LPS vs. LPS+Hyperglycemia+HUCMSCS | 0.11 | 0.343 | 0.001* | 0.007* | 0.004* | 0.001* | 0.001* |
| LPS vs. LPS+ Hyperglycemic +HUCMSCS+NEP | 0.001* | 0.001* | 0.001* | 0,002 | 0.001* | 0.001* | 0.001* |
| Hyperglycemia vs. LPS+Hyperglycemia | 0.001* | 0.001* | 0.03* | 0.739 | 0.015* | 0.565 | 0.887 |
| Hyperglycemia vs. LPS+Hyperglycemia+NEP | 0.09 | 0.121 | 0.514 | 0.001* | 0.001* | 0.001* | 0.003* |
| Hyperglycemia vs. LPS+Hyperglycemia+HUCMSCS | 0.001* | 0.001* | 0.001* | 0.001* | 0.001* | 0.001* | 0.001* |
| Hyperglycemia vs. LPS+ Hyperglycemic +HUCMSCS+NEP | 0.001* | 0.001* | 0.001* | 0.001* | 0.001* | 0.001* | 0.001* |
| LPS+Hyperglycemia vs. LPS+Hyperglycemia+NEP | 0.001* | 0.001* | 0.001* | 0.001* | 0.001* | 0.001* | 0.001* |

***Information:** there was significant different between groups analyzed with Tukey HSD at p<0.05.
